# Effects of Herbal Mouthwashes on Plaque and Inflammation Control for Patients with Gingivitis: A Systematic Review and Meta-Analysis of Randomised Controlled Trials

**DOI:** 10.1155/2020/2829854

**Published:** 2020-01-20

**Authors:** He Cai, Junyu Chen, Nirmala K. Panagodage Perera, Xing Liang

**Affiliations:** ^1^State Key Laboratory of Oral Diseases, National Clinical Research Center for Oral Diseases, West China Hospital of Stomatology, Sichuan University, No. 14, Section 3, South Renmin Road, Chengdu 610041, China; ^2^Department of Prosthodontics, West China Hospital of Stomatology, Sichuan University, No. 14, Section 3, South Renmin Road, Chengdu 610041, China; ^3^Department of Orthopaedics, Rheumatology, and Musculoskeletal Sciences, Centre for Sport, Exercise and Osteoarthritis Research Versus Arthritis, University of Oxford, Windmill Road, Oxford OX3 7LD, UK; ^4^Kennedy Institute of Rheumatology, University of Oxford, Roosevelt Drive, Oxford OX3 7FY, UK; ^5^La Trobe Sports and Exercise Medicine Research Centre, College of Science, Health and Engineering, La Trobe University, Bundoora, VIC 3086, Australia

## Abstract

**Objective:**

The aim of this study was to evaluate the overall effects of herbal mouthwashes as supplements to daily oral hygiene on plaque and inflammation control compared with placebos and chlorhexidine (CHX) mouthwashes in the treatment of gingivitis.

**Methods:**

PubMed, EMBASE, Cochrane Database of Systematic Reviews, Cochrane Central Register of Controlled Trials, and grey literature databases were searched. Only randomised controlled trials (RCTs) comparing herbal mouthwashes with placebos or CHX in the daily oral hygiene of patient with gingivitis were included to compare the effect of different mouthwashes on plaque and inflammation control.

**Results:**

A total of 13 studies satisfied the eligibility criteria, and 11 studies were included in meta-analyses. Significant differences were observed in favour of herbal mouthwashes compared with placebos in both plaque- and inflammation-related indices (Quigley-Hein Plaque Index, QHPI: WMD = −0.61, 95% CI (−0.80, −0.42), *P* < 0.001; Gingival Index, GI: −0.28 (−0.51, −0.06), *P*=0.01; Modified Gingival Index, MGI: −0.59 (−1.08, −0.11), *P*=0.02; Gingival Bleeding Index, GBI: −0.06 (−0.09, −0.04), *P* < 0.001). No significant difference was found between herbal and CHX mouthwashes.

**Conclusions:**

Herbal mouthwashes have potential benefits in plaque and inflammation control as supplements to the daily oral hygiene of patients with gingivitis. Although no difference was observed between herbal and CHX mouthwashes in the selected studies, further high-quality RCTs are needed for more firm support before advising patients with gingivitis about whether they can use herbal mouthwashes to substitute for CHX mouthwashes or not (*PROSPERO registration number*: CRD42019122841).

## 1. Introduction

Gingivitis, which has a direct association with dental plaque [1, 2], affects the oral health of 70%–100% of the population across the world [3–5]. Gingivitis is reversible through plaque control; however, it may progress with inadequate oral care and eventually affect the entire periodontal attachment apparatus of the involved teeth, resulting in further harmful consequences, such as periodontitis, tooth loss, and worse quality of life [6]. Thus, effective plaque control plays a crucial role in resolving and preventing gingivitis and related conditions [7].

Currently, toothbrushing is the most popular self-performed oral hygiene method to mechanically remove dental plaque. However, this mechanical approach by most individuals is often not sufficiently effective [8], suggesting that a chemical plaque control by mouthwashes could be beneficial as a supplement to daily oral care [9]. Chlorhexidine (CHX), a broad-spectrum antiseptic, is considered as the gold standard for chemical dental plaque control [2], but CHX mouthwashes can lead to the staining of teeth and tongue, taste disturbance, and adverse effects on the oral mucosa after prolonged use [10, 11]. Those undesirable side effects limit the long-term use and the patient acceptability of CHX mouthwashes. Thus, the search for alternatives continues, and the focus shifted toward biogenic agents.

Herbal medicines, derived from botanical sources, have been applied in dentistry for a long history to inhibit microorganisms, reduce inflammation, soothe irritation, and relieve pain [12–14]. It has been recently reported that a considerable number of herbal mouthwashes have achieved encouraging results in plaque and gingivitis control [15, 16]. Herbal mouthwashes are designed and prepared with extracts and essential oils from phytotherapeutic plants, containing a mixture of active agents such as catechins, tannins, and sterols [17, 18]. The mixture of natural compounds inside the herb- or plant-derived substances usually performs gentle remedial effects. Compared with the antimicrobial mechanisms by synthetic chemicals, herbal mouthwashes can have additional anti-inflammatory and antioxidant properties, which could further benefit gingival health [19].

Numerous herbal mouthwashes have been introduced and tested; however, the results of existing literature are inconsistent regarding the clinical effects of herbal mouthwashes on both dental plaque and gingival inflammation control compared with placebo or CHX [20–22], and there is a scarcity of meta-analytical evidence highlighting the overall effects of herbal mouthwashes as adjuncts to the daily self-performed oral hygiene of patients with gingivitis. Without this information, it is not possible to provide comprehensive evidence-based advice to the patients and practitioners. Therefore, the aim of this study was to conduct a systematic review and a meta-analysis of randomised controlled trials (RCTs) only to compare the overall effects of herbal mouthwashes as supplements to the daily oral hygiene on both plaque and inflammation control with either negative placebos or CHX mouthwashes in the treatment of gingivitis.

## 2. Materials and Methods

The current systematic review was reported following the Preferred Reporting Items for Systematic Reviews and Meta-analyses (PRISMA) guidelines [23]. A detailed protocol was developed a priori and registered in the International Prospective Register of Systematic Reviews (http://www.crd.york.ac.uk/PROSPERO/) (registration number: CRD42019122841).

### 2.1. Participants-Interventions-Comparisons-Outcomes-Study Design (PICOS) Question

This systematic review was performed to answer the focused question “*How are the effects of herbal mouthwashes for plaque and inflammation control as a supplement to daily oral hygiene when compared to placebos or CHX-containing mouthwashes among patients with gingivitis?*” according to the following elements:

  Participants: systematically healthy participants with gingivitis
  Interventions: the application of herbal mouthwashes from botanical sources
  Comparisons: the application of negative placebos without any active agents or positive CHX-containing mouthwashes
  Outcomes: the clinical effects of mouthwashes as a supplement to daily oral hygiene (i.e., toothbrushing) on plaque and inflammation control
  Study design: RCTs only

### 2.2. Eligibility Criteria

Based on the PICOS question, a study must fulfil the following inclusion criteria:

- Studies with participants who had gingivitis and were otherwise systemically healthy during the test period
- The intervention group(s) must use a mouthwash containing extract(s) or essential oil(s) from plants
- The comparison group(s) must comprise a negative placebo solution or a CHX-containing mouthwash
- All the interventions should be applied as a supplement to daily self-performed oral hygiene routine
- Studies should include at least one plaque- or gingivitis-related indices as an outcome measure
- RCTs

It should be noted that only RCTs were included to synthesise high-quality evidence which would then enable inferences to be drawn with confidence. Studies that had participants with periodontitis or participants who were undertaking orthodontic treatment; studies which only reported outcomes relating to the specific oral bacteria (such as the *Streptococcus mutans* count); and *in vitro*, animal or cadaver studies, case studies, letters, and historical reviews were excluded. Further, to replicate the “real-life” circumstances of patients, studies with professional mechanical prophylaxis during or in three months before the trials were excluded.

### 2.3. Information Sources and Literature Search

PubMed, EMBASE, Cochrane Database of Systematic Reviews (CDSR), and Cochrane Central Register of Controlled Trials (CENTRAL) databases were systematically searched for relevant articles written in English, from inception to 22 February 2019. The following search strategy was established based on the PICOS framework to gain a highly sensitive group of descriptors, combining the population- and intervention-related MeSH and free text words.

1.  Participants–“Gingivitis” [MeSH] OR “Gingival Hemorrhage” [MeSH] OR “Dental Plaque” [MeSH] OR gingivit^*∗*^ OR plaque^*∗*^ OR biofilm^*∗*^ OR microorganism^*∗*^ OR microflora^*∗*^ OR “gingival pocket” OR “gingival pockets” OR “pseudo pocket” OR “pseudo pockets” OR pseudopocket^*∗*^ OR ((gingiv^*∗*^ OR papilla^*∗*^ OR sulc^*∗*^) AND (hemorrha^*∗*^ OR bleed^*∗*^ OR inflam^*∗*^))
2. Interventions–(“Botany” [MeSH] OR “Phytotherapy” [MeSH] OR “Plant Preparations” [MeSH] OR “Plants, Medicinal” [MeSH] OR “Medicine, Traditional”[MeSH] OR herb^*∗*^ OR plant^*∗*^ OR extract^*∗*^ OR phyto^*∗*^ OR botan^*∗*^ OR “essential oil” OR “essential oils” OR tea^*∗*^ OR ayurved^*∗*^ OR kampo^*∗*^ OR shaman^*∗*^ OR ((medicine^*∗*^ OR formula^*∗*^) AND (tradition^*∗*^ OR Chinese OR African OR Tibetan OR Mongolian OR Japanese OR Indian OR Korean OR Arabic OR Unani))) AND (“Mouthwashes” [MeSH] OR mouthrinse^*∗*^ OR mouthwash^*∗*^ OR collut^*∗*^ OR gargle^*∗*^ OR rinse^*∗*^ OR wash^*∗*^)
3. Participants AND Interventions–(1) AND (2)

For “grey” literature, the ClinicalTrials.gov and the International Clinical Trials Registry Platform were searched for unpublished clinical studies or registries; the ProQuest Dissertation Abstracts and Thesis database was searched for related dissertations and theses; the Conference Proceedings Citation Index-Science was searched for conference proceedings; additionally, some other online resources such as the System for Information on Gray Literature in Europe database were also searched as a supplement. Subsequently, a manual search was carried out based on the reference lists of selected trials and related reviews.

### 2.4. Study Selection

Duplicates were removed, and the titles and abstracts were screened by two independent reviewers (HC and JC) against the inclusion/exclusion criteria. After the initial screening, full texts of the potentially relevant articles were retrieved. When the articles [16, 24–26] were unavailable online, then the corresponding authors or the editors of the journals were contacted to request full-text articles, and two full-text articles [16, 24] were received. Once all the full-text articles were collected, they were independently screened by the same two authors. If any article failed to meet a single criterion, then it was immediately excluded. If there was disagreement regarding inclusion/exclusion of a study, this was resolved through discussion or by a third reviewer (XL).

### 2.5. Data Collection and Data Items

A standard data extraction sheet was developed *a priori*, and the data were extracted and cross-checked for accuracy by two independent reviewers (HC and JC). Detailed data pertaining to author; year of publication; sample size; age range; number of males and smokers; active pharmaceutical ingredients, detailed dosage, and putative chemical properties of applied herbal mouthwashes; comparisons; relevant plaque- and/or gingivitis-related measures; follow-up; loss to follow-up; and effects and side effects of herbal mouthwashes reported in the selected studies were extracted.

To accurately extract the data that were presented in graphs, WebPlotDigitizer [27] (http://automeris.io/WebPlotDigitizer, A. Rohatgi, Austin, Texas, United States), an online image extraction tool, was used.

### 2.6. Risk of Bias in Individual Trials

The risk of bias was independently graded by two reviewers (HC and JC) using the Cochrane Collaboration's tool for assessing risk of bias [28]. The risk of bias within studies was estimated as “low,” “unclear,” or “high” through seven aspects of criteria including random sequence generation, allocation concealment, blinding of participants and personnel, blinding of outcome assessment, incomplete outcome data, selective reporting, and other bias. The RevMan version 5.3 (The Cochrane Collaboration, Copenhagen, Denmark) was used.

### 2.7. Summary Measures

The clinical outcomes were mainly evaluated with Plaque Index (PI) [29] and Turesky modification of Quigley-Hein Plaque Index (QHPI) [30] for supragingival plaque, and Gingival Index (GI) [31] and Modified Gingival Index (MGI) [32] were used for visual signs of gingival inflammation. Gingival bleeding was also reported in a few studies as another sign for gingival inflammation via varied measures, such as Bleeding Index (BI) [33], Sulcus Bleeding Index (SBI) [34], and Gingival Bleeding Index (GBI) [35]. Means and standard deviations (SDs), as well as data in other formats (e.g., standard errors, medians, and interquartile ranges) reported for these measures, were collected for further meta-analyses.

### 2.8. Synthesis of Results

To compare the antimicrobial and anti-inflammatory properties of herbal mouthwashes to negative placebos and positive “gold standard” CHX-containing mouthwash and to increase the accuracy of the overall estimate of the effect size, the data were pooled from individual studies with the same comparison and outcome measure to perform a meta-analysis. Due to the random allocation in individual studies, the mean and SD of each index at the endpoint of study in each group (herbal, placebo, and CHX) were obtained to estimate the weighted mean differences (WMDs) and their 95% confidential intervals (95% CIs), comparing the effects between different mouthwashes. Some studies reported other data formats and when possible were converted into the desired format (i.e., means and SDs) to include in the analyses [36]. The missing SDs in some articles were estimated by the average of SDs from other studies in the same meta-analysis [37]. The extracted data were analysed using RevMan version 5.3 software (The Cochrane Collaboration, Copenhagen, Denmark). Heterogeneity across trials was investigated using the Chi-squared test and *I*^2^ statistics [38]. Considering the sample size of individual studies and the heterogeneity across trials, either a fixed effects model or a random effects model [39] was applied. No subgroup analysis or metaregression was performed due to the limited number of eligible studies.

### 2.9. Risk of Bias across Studies

Due to the limited number of included publications, the publication bias across studies was investigated using Egger's test by Stata SE release 15 (StataCorp LP, College Station, TX) [40].

## 3. Results

### 3.1. Study Selection

**Figure 1:**
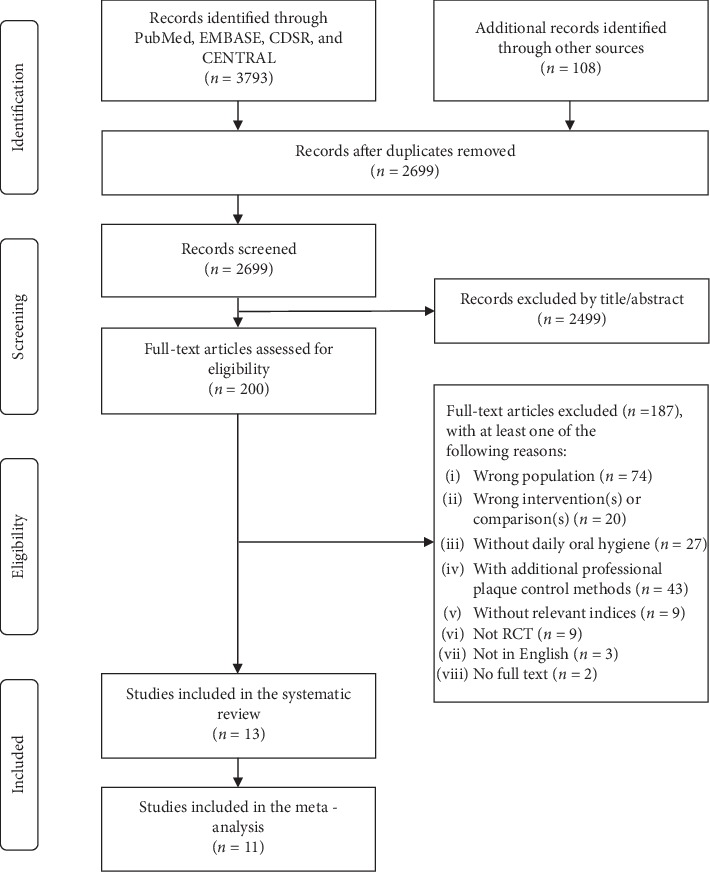
Preferred Reporting Items for Systematic Reviews and Meta-Analyses (PRISMA) flow diagram for study selection (CDSR, Cochrane Database of Systematic Reviews; CENTRAL, Cochrane Central Register of Controlled Trials; RCT, randomised controlled trial).

The initial search identified 2,699 studies, in which 13 studies [17, 18, 20–22, 24, 41–47] based on independent cohorts satisfied the inclusion criteria (Figure 1). Of these, 11 studies [18, 20–22, 24, 41–46] were included in the meta-analyses.

### 3.2. Study Characteristics

Table 1 summarised the relevant characteristics of 13 qualified RCTs. The clinical effects compared between herbal mouthwashes and negative placebos were evaluated in seven RCTs [17, 18, 20–22, 24, 43] with a total of 612 participants. Eight studies [20, 22, 41, 42, 44–47] with 287 participants evaluated the effects of herbal and CHX mouthwashes. All the included studies were carried out after the year 2000, and nine [17, 18, 20, 22, 24, 42–44, 47] were performed after the year 2010, indicating that the popularity of the herbal mouthwashes might be growing. Six studies [22, 24, 42, 43, 45, 46] had a balanced gender ratio, and five studies [18, 20, 44–46] excluded all the smokers in their samples.

The details of interventions were provided in Table 1. All the herbal mouthwashes in selected studies were made of natural extracts and essential oils derived from herbs or plants. The most commonly used herb- or plant-derived substance reported in the selected studies was green tea extract [17, 21, 42, 47], followed by neem [20, 42, 46] and marigold [18, 21, 22]. The commercial essential oil-containing mouthwashes were also used in some studies [24, 43, 46], which usually consist of eucalyptol, menthol, methyl salicylate, and thymol. In these selected studies, all the natural ingredients in the herbal mouthwashes were expected to have antibacterial, anti-inflammatory, or antioxidant properties. However, few possible active compounds and relevant molecular mechanisms inside the antiplaque/antigingivitis effects of herbal extracts were studied in these clinical trials. The follow-ups within studies between herbal and CHX were always less than four weeks as the long-term use of CHX mouthwashes is not recommended due to its side effects [48], while the studies between herbal mouthwashes and placebos could have a longer follow-up up to 24 weeks [43]. Most herbal mouthwashes were demonstrated to have potential benefits in reducing plaque and inflammation for gingivitis patients as supplements to daily oral hygiene.

Four studies [17, 21, 24, 46] addressed the side effects during herbal mouthwashes rinsing and reported some degree of hypogeusia [24], lightheadedness [21], and an unpleasant taste [17, 46]. In the other nine studies, five [18, 22, 41–43] reported that herbal mouthwashes were well tolerated with no side effects, and the remaining studies [20, 44, 45, 47] made no comment on side effects or adverse events.

### 3.3. Risk of Bias within Studies

**Figure 2:**
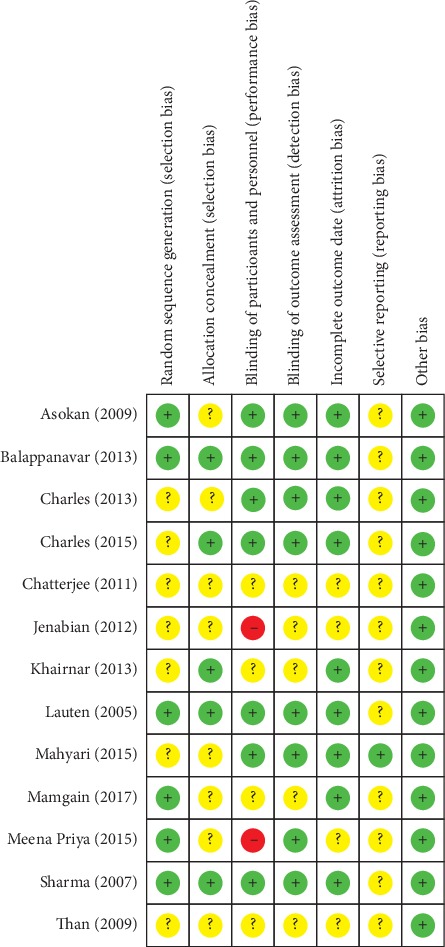
Risk of bias summary for reviewing authors' judgements about each risk of bias item for each included study. Green stands for low risk of bias, yellow represents unclear risk of bias, and red indicates high risk of bias.

Eleven studies [18, 20–22, 24, 41–46] were of low and unclear risk of bias, and two studies [17, 47] included a high risk of bias (Figure 2). Six studies [21, 41, 42, 44, 46, 47] had a low risk of bias for random sequence generation, and five [18, 21, 24, 42, 46] provided clear information in terms of allocation concealment. Two studies [17, 47] failed to blind the participants and personnel (Figure 2), which introduced a high risk of bias and were then excluded from the meta-analyses to raise the quality of evidence in this study [49]. Thus, all the studies included in meta-analyses were without any high risk of bias.

### 3.4. Results of Individual Studies and Synthesis of Results

The focus of this study was mainly to compare the overall clinical effects between herbal mouthwashes and placebo or CHX mouthwashes on plaque and inflammation control. Five included studies [18, 20, 22, 24, 43] showed significantly greater reductions in plaque, gingivitis, and bleeding indices after the use of herbal mouthwashes compared with placebos, while two studies [17, 21] reported that no difference was observed between herbal mouthwashes and placebos. Five studies [20, 22, 41, 44, 45] revealed that there was no difference between herbal mouthwashes and CHX for plaque or inflammation control, and two studies [42, 47] even showed greater reductions in gingival inflammation after the intervention of herbal mouthwashes. However, in another study, no significant changes were observed in either the plaque index or gingivitis index after the use of herbal mouthwash, while CHX showed significant effects on both the two indices [46].

#### 3.4.1. Meta-Analyses Comparing Herbal Mouthwashes and Placebos

When herbal mouthwashes and placebo were compared by meta-analysis commands, five studies [18, 21, 22, 24, 43] were included for QHPI analysis, three [18, 20, 21] for GI, three [22, 24, 43] for MGI, and three [22, 24, 43] for GBI.

**Figure 3:**
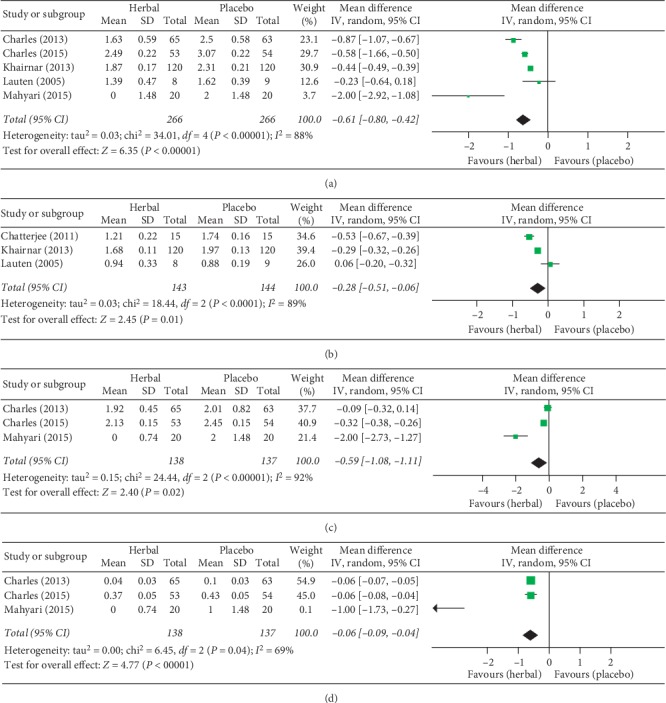
Forest plots comparing the clinical effects by specific indices between herbal mouthwashes and placebos. Comparison for (a) Turesky modification of Quigley-Hein Plaque Index (QHPI), (b) Gingival Index (GI), (c) Modified Gingival Index (MGI), and (d) Gingival Bleeding Index (GBI).

Significant differences were observed in all these analyses (QHPI, GI, MGI, and GBI) in favour of herbal mouthwashes rather than placebos (Figure 3). The mean QHPI at the endpoint of follow-up was significantly lower after the use of herbal mouthwashes (*herbal to placebo*: QHPI: WMD = −0.61, 95% CI (−0.80, −0.42), *P* < 0.001). As to the effects on gingival inflammation-related indices, herbal mouthwashes had a significantly higher decrease in GI (−0.28 (−0.51, −0.06), *P*=0.01), MGI (−0.59 (−1.08, −0.11), *P*=0.02), and GBI (−0.06 (−0.09, −0.04), *P* < 0.001) compared to placebos. Substantial heterogeneity was observed in those meta-analyses (QHPI: Chi^2^ = 34.01 > 4, *I*^2^ = 88%; GI: Chi^2^ = 18.44 > 2, *I*^2^ = 89%; MGI: Chi^2^ = 24.44 > 2, *I*^2^ = 92%; GBI: Chi^2^ = 6.45 > 2, *I*^2^ = 69%).

#### 3.4.2. Meta-Analyses Comparing Herbal and CHX Mouthwashes

**Figure 4:**
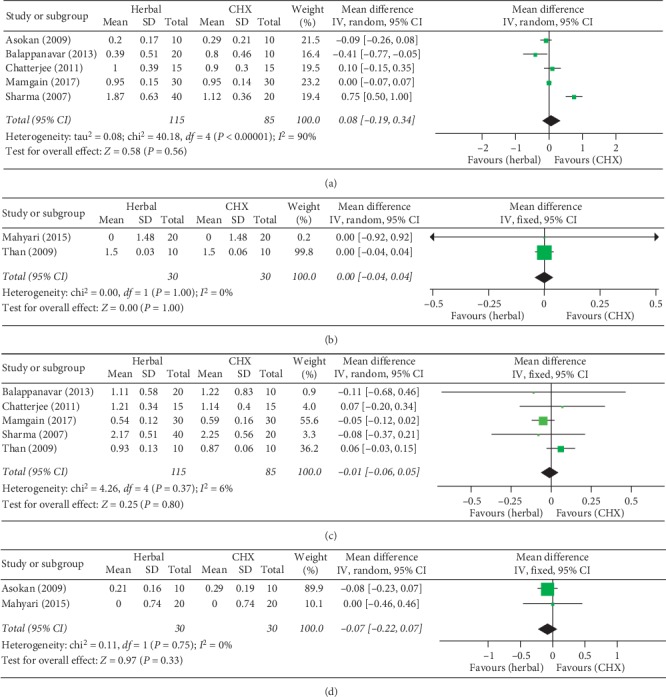
Forest plots comparing the clinical effects by specific indices between herbal and chlorhexidine (CHX) mouthwashes. Comparison for (a) Plaque Index (PI), (b) Turesky modification of Quigley-Hein Plaque Index (QHPI), (c) Gingival Index (GI), and (d) Modified Gingival Index (MGI).

When herbal and CHX mouthwashes were compared, five studies [20, 41, 42, 44, 46] were included for PI analysis, two [22, 45] for QHPI, five [20, 42, 44–46] for GI, and two [22, 41] for MGI. There was no significant difference between herbal and CHX mouthwashes regarding the four clinical indices (Figure 4) (PI: 0.08(−0.19, 0.34), *P*=0.56; QHPI: 0.00(−0.04, 0.04), *P*=1.00; GI: −0.01 (−0.06, 0.05), *P*=0.80; MGI: −0.07(−0.22, 0.07), *P*=0.33) (Figure 4). Heterogeneity varied in the analyses of different outcomes (PI: Chi^2^ = 40.18 > 4, *I*^2^ = 90%; QHPI: Chi^2^ = 0.00 < 1, *I*^2^ = 0%; GI: Chi^2^ = 4.26 > 4, *I*^2^ = 6%; MGI: Chi^2^ = 0.11 < 1, *I*^2^ = 0%).

### 3.5. Risk of Bias across Studies

Publication bias assessments using Egger's test (*herbal to placebo*: QHPI: coefficient = −2.49 95% CI (−7.81, 2.83), *P*=0.233; GI: −0.18 (−47.40, 47.03), *P*=0.968; MGI: −2.02 (−49.49, 45.44), *P*=0.684; GBI: −2.22 (−13.60, 9.15), *P*=0.244; *herbal to CHX*: PI: 1.18 (−8.19, 10.55), *P*=0.715; QHPI: *n* = 2, Egger's test could not be performed; GI: 0.01 (−2.92, 2.94), *P*=0.990; MGI: *n* = 2, Egger's test could not be performed) indicated that there was no significant evidence of publication bias among the included articles of the eight meta-analyses. However, those were for reference only due to the insufficient literatures included.

## 4. Discussion

The present study is the first systematic review and meta-analysis to investigate the clinical effects of herbal mouthwashes as supplements to the self-performed oral hygiene of patients with gingivitis. It is found that herbal mouthwashes are effective in reducing plaque and gingivitis, and there was no difference between herbal and CHX mouthwashes. The effects of herbal mouthwashes were intended to be studied in a “real-life” condition, and hence the professional dental prophylaxis was excluded from this study to make sure that all the involved participants could begin the treatment with their normal existing plaque deposits and gingival inflammation.

Herbal medicines used in oral hygiene is popular due to their antimicrobial, anti-inflammatory, and antioxidant properties [50, 51]. There is a dynamic and rapid accumulation of clinical trials showing that mouthwashes containing herbal exacts, such as *Camellia sinensis* (green tea) and *Azadirachta indica* (neem), and essential oils can probably be an effective treatment to reduce dental plaque and gingival inflammation [18, 52, 53]. Based on the evidence of relevant RCTs, it is indicated that herbal mouthwashes have potential antiplaque and antigingivitis effects compared with placebos. The natural botanical products seem to be a promising field for the treatment of gingivitis, and further studies are needed to seek for more effective herbal mouthwashes with either the traditional medicine formulations or new herbal preparations. However, the exact mechanisms of the antiplaque and antigingivitis actions of herbal mouthwash treatments were not clear due to the inherent complex nature of botanical products. The possible mechanisms can probably be that the natural compounds, such as catechins, tannins, sterols, and oils, may inhibit the growth and adhesion of oral microorganisms and subside inflammatory cytokines such as interleukin-6 and tumour necrosis factor-*α* [17, 18, 41, 54]. Also, herbal mouthwashes may protect the gingiva from infection and inflammation by their antioxidant property [41]. Further studies are required to clarify the biological mechanisms inside the antiplaque and antigingivitis effect of those herbal mouthwashes. With the literature indicating that herbal mouthwashes are superior to placebo as a supplement to daily oral hygiene [18, 52, 53], practitioners would be better informed about the potential choices if the comparisons could be conducted directly between herbal mouthwashes and one “good standard” antiseptic.

CHX has been considered as the primary agent for chemical plaque control for more than four decades. However, CHX has several side effects, such as tooth staining [55]. CHX-containing mouthwashes may result in the local colour precipitation between tooth-bound CHX and the chromogens in the daily diet, causing unpleasant brownish staining or discolouration [56, 57]. Also, there can be an idiosyncratic reaction of oral mucosal desquamations and soreness after the use of CHX [55]. Mouthwashes containing herbal extracts were investigated to have less or even no staining effect [58, 59]. Based upon the existing evidence, no significant differences in the effects on plaque or gingivitis were observed between herbal and CHX mouthwashes. Notably, some specific herbal mouthwashes, such as neem and green tea, were reported to have an even better effect than CHX on gingiva [42]. Therefore, based on the findings of this current study, herbal mouthwashes seem to be possible alternatives to CHX-containing mouthwashes as part of the daily oral hygiene of patients with gingivitis, especially for the long-term use. However, due to the limited studies included in this review, it is not sufficient to advise patients with gingivitis about whether they can use herbal mouthwashes to substitute for CHX mouthwashes or not. Further high-quality RCTs are needed to get more firm evidence for clinical decision-making.

The studies selected for this review also reported a few side effects during herbal mouthwash rinsing, such as hypogeusia, bitter taste, and lightheadedness in individual participants. However, the relevant evidence was insufficient, and the safety of herbal mouthwash still warrants more careful examination.

The evidence of this study should be viewed considering the limitations of the study. High heterogeneity was observed in some analyses, possibly due to the variation in the baseline indices across studies. However, as all the included studies were RCTs, the baseline indices of test group and control group within individual trials were estimated to be comparable with each other; hence, the specific indices at the endpoint after different interventions were collected and synthesised for the comparisons between the clinical effects of different mouthwashes. In this way, the variation of the baseline indices across trials might make little influence on the main aim of this meta-analysis, although it could be one of the reasons for heterogeneity. Also, the varied age range, percentage of males and smokers, active ingredients in herbal mouthwashes, and follow-up across studies had not achieved complete consistency, which might contribute to the high heterogeneity. Unfortunately, it was unable to detect the exact sources of heterogeneity within the review due to the limited number of included studies. Thus, further studies are needed for metaregression and subgroup analysis. The loss to follow-up is a very important aspect of a clinical trial, as the incomplete follow-up in RCTs may affect the validity of data and increase the bias [60, 61]. However, as some included studies did not provide clear information in respect of the loss to follow-up, it was not possible to verify the validity of the evidence within individual studies. Future studies should clearly report the number of participants who enter and exit the studies to enable the calculation of loss to follow-up and state findings accordingly. Moreover, two RCTs [17, 47] were found with a high risk of bias in blinding of participants and personnel by reporting a single-blinded trial without participants blinded, which might result in a drop of the internal validity of the study [49]. To minimise the likelihood of performance bias and raise the quality of evidence within this meta-analysis, the two studies were excluded from meta-analysis and were described for characters only. Additionally, the limited number of included studies implied that there might be a lack of sufficient evidence and robust assessments of publication biases for most of the outcomes, compromising the validity of analysis to some extent. Therefore, further high-quality RCTs are needed for stronger support of clinical decision-making.

## 5. Conclusions

Within the limitations of this current study, it can be concluded that herbal mouthwashes have potential benefits in plaque and inflammation control as supplements to the daily oral hygiene of patients with gingivitis. Although no difference was observed between herbal and CHX mouthwashes in the selected studies, further high-quality RCTs are needed for more firm support before advising patients with gingivitis about whether they can use herbal mouthwashes to substitute for CHX mouthwashes or not. Also, the botanical products can be considered as a promising field for the treatment of gingivitis, and it warrants further research to seek for more effective herbal mouthwashes with either the traditional medicine formulations or new herbal preparations.

## Figures and Tables

**Table 1:** Relevant characteristics of the included randomised controlled trials.

Study	Sample size	Age range	Males (%)	Smokers (%)	Herbal mouthwash(es)		Comparison(s)^#^	Relevant clinical measures	Effects	Follow-up	Loss (%)	Side effects
					Active pharmaceutical ingredients and dosage^#^	Putative chemical properties						
Asokan et al. [41]	20	16–18	20 (100%)	NR	100% *Sesamum indicum* (sesame) oil, a tablespoon (? mL), 60 s, once daily before brushing	Antioxidant, antibacterial, anti-inflammatory, and saponification properties	0.12% CHX, ? mL, 60 s, once daily before brushing	PI, MGI	Significant reductions in plaque and gingivitis were observed in both the herbal and CHX groups, and there was no difference between them.	10 days	0 (0%)	No side effects

Balappanavar et al. [42]	30	18–25	15 (50%)	NR	(i) 2% azadirachta indica (neem) extract, 15 mL, 30 s, twice daily after brushing; (ii) 0.5% camellia sinensis (green tea) extract, 15 mL, 30 s, twice daily after brushing	Neem: antimicrobial and anti-inflammatory properties; Green tea: antioxidant, anti-inflammatory, and antibacterial properties	0.2% CHX, 15 mL, 30 s, twice daily after brushing	PI, GI	Significant reductions in plaque were observed in all groups and the highest being in CHX groups; neem and tea shown comparative effects on gingivitis better than CHX.	2-3 weeks^†^	0 (0%)	No side effects

Charles et al. [43]	139	18–61	61 (44%)	17 (12%)	Listerine® essential oil-containing mouthwash, eucalyptol, menthol, methyl salicylate, and thymol, 20 mL, 30 s, twice daily	Antimicrobial, antiplaque, and antigingivitis properties	Placebo, 20 mL, 30 s, twice daily	QHPI, MGI, GBI	Significant greater reductions in plaque, gingivitis, and bleeding were observed in herbal mouthwash group compared with the placebo group.	24 weeks	11 (8%)	No side effects

Charles et al. [24]	165	18–72	69 (42%)	20 (12%)	Listerine® cool mint essential oil-containing mouthwash, 0.092% eucalyptol, 0.042% menthol, 0.060% methyl salicylate and 0.064% thymol, 20 mL, 30 s, twice daily after brushing	Antimicrobial, antiplaque, and antigingivitis properties	Placebo, 20 mL, 30 s, twice daily after brushing; 0.075% CPC, 20 mL, 30 s, twice daily after brushing	QHPI, MGI, GBI	Significantly greater reductions in plaque, gingivitis, and bleeding were observed in herbal mouthwash group compared with placebo and CPC groups.	4 weeks	3 (2%)	Hypogeusia

Chatterjee et al. [20]	45	18–65	NR	0 (0%)	0.19% *Azadirachta indica* (neem) extract, 15 mL, 60 s, twice daily	Antimicrobial and anti-inflammatory properties	Placebo, 15 mL, 60 s, twice daily 0.2% CHX, 15 mL, 60 s, twice daily 0.2% CHX, 15 mL, 60 s, twice daily	PI, GI, BI	Significantly greater reductions in plaque, gingivitis, and bleeding were observed in herbal mouthwash group compared with the placebo group, and there was no difference between herbal and CHX mouthwashes.	3 weeks	0 (0%)	NR

Jenabian et al. [17]^*∗*^	50	14–16	0 (0%)	NR	5% *Camellia sinensis* (green tea) extract, 5 mL, 30 s, twice daily	Antioxidant, anti-inflammatory, and antibacterial properties	Placebo, 5 mL, 30 s, twice daily	PI, GI, MPBI	There was no difference between herbal mouthwash and placebo; however, the changing patterns of indices were significantly different.	6 weeks	NR	Unpleasant taste

Khairnar et al. [18]	240	20–40	NR	0 (0%)	25% *Calendula officinalis* (marigold) tincture, 8 mL, ? s, twice daily	Antibacterial, antifungal, anti-inflammatory, antioxidant, and immunomodulatory properties	Placebo, 8 mL, ? s, twice daily	QHPI, GI, SBI	Significantly greater reductions in plaque, gingivitis, and bleeding were observed in herbal mouthwash group compared with the placebo group.	3 months	0 (0%)	No side effects

Lauten et al. [21]	20	≥18	3 (15%)	NR	0.67% *Melaleuca alternifolia* (melaleuca) oil, 0.33% *Leptospermum scoparium* (manuka) oil, 1% *Calendula officinalis* (marigold) extract, and 0.5% *Camellia sinensis* (green tea) extract, 15 mL, 30 s, twice daily	Antimicrobial and anti-inflammatory properties	Placebo, 15 mL, 30 s, twice daily	QHPI, GI	There was no difference between herbal mouthwash and placebo.	3 months	3 (15%)	Lightheadedness

Mahyari et al. [22]	60	18–65	25 (42%)	NR	5% *Calendula officinalis* (marigold) extract, 5% *Zingiber officinale* (ginger) extract and 5% *Rosmarinus Officinalis* L. (rosemary) extract, 10 mL, 30 s, twice daily	Antibacterial, antioxidant, and anti-inflammatory properties	Placebo, 10 mL, 30 s, twice daily 0.2% CHX, 10 mL, 30 s, twice daily	QHPI, MGI, GBI	Significantly greater reductions in plaque, gingivitis, and bleeding were observed in herbal mouthwash group compared with the placebo group, and there was no difference between herbal and CHX mouthwashes.	2 weeks	0 (0%)	No side effects

Mamgain et al. [44]	60	≥18	NR	0 (0%)	3 g *Elettaria cardamomum* (ela) seed powder mixed in 100 mL triphala (equal parts of *Emblica officinalis*, *Terminalia chebula*, and *Terminalia belerica*), ? mL, 60 s, twice daily	Antiplaque, antigingivitis, and antihalitosis properties	?% CHX, ? mL, 60 s, twice daily	PI, GI	Significant reductions in plaque and gingivitis were observed in both the herbal and CHX groups, and there was no difference between them.	3 weeks	NR	NR

Meena Priya et al. [47]^*∗*^	30	18–24	NR	NR	5% *Camellia sinensis* (green tea) extract, ? mL, 30 s, after brushing	Antioxidant, anti-inflammatory, and antibacterial properties	?% CHX, ? mL, 30 s, after brushing	QHPI, GI, BI′	Significant reductions in plaque, gingivitis, and bleeding were observed in both the herbal and CHX groups, and there was a significant greater reduction of bleeding in herbal group compared with CHX.	1 month	NR	NR

Sharma et al. [46]	80	12–20	42 (53%)	0 (0%)	(i) 1% *Azadirachta indica* (neem) extract, 10 mL, 60 s, twice daily after brushing; (ii) Listerine® essential oil-containing mouthwash, 10 mL, 60 s, twice daily after brushing	Neem: antimicrobial and anti-inflammatory properties; Essential oil: antiplaque and mild anti-inflammatory properties	0.2% CHX, 10 mL, 60 s, twice daily after brushing; 1% PVP-I Povidone iodine, 10 mL, 60 s, twice daily after brushing	PI, GI	No significant reductions were observed in the neem groups, and there was a significant reduction in plaque and gingivitis in essential oil, CHX, PVP-I groups.	2 weeks	0 (0%)	Bitter taste

Than et al. [45]	20	17–20	10 (50%)	0 (0%)	0.2% *Ixora coccinea Linn.* (Ponna yeik) extract, 20 mL, 60 s, twice daily	Antibacterial and anti-inflammatory (relieving the gum swelling in palate) properties	0.2% CHX, 20 mL, 60 s, twice daily	QHPI, GI, SBI	Significant reductions in plaque, gingivitis, and bleeding were observed in both the herbal and CHX groups, and there was no difference between them.	4 weeks	NR	NR

*Note.* NR, not reported; CHX, chlorhexidine; CPC, cetylpyridinium chloride; PVP-I, povidone iodine; Plaque-related indices: PI, Plaque Index by Sillness and Loe; QHPI: Turesky modification of Quigley-Hein Plaque Index by Turesky et al.; Gingival inflammation-related indices: *Gingivitis indices*: GI, Gingival Index by Loe and Silness; MGI, Modified Gingival Index by Lobene et al.; *Bleeding indices*: BI, Bleeding Index by Sillness and Loe; BI′, Bleeding Index by Ainamo and Bay; GBI: Gingival Bleeding Index by Saxton and van der Ouderaa; SBI, Sulcus Bleeding Index by Mühlemann and Son; MPBI, Modified Papillary Bleeding Index by Barnett et al.; BOMP: Bleeding on Marginal Probing by Vander Weijden et al.; ^*∗*^Those two studies marked with grey were excluded in the meta-analyses due to the high risk of bias within studies. ^#^The unreported details of mouthwashes in selected studies were described with question marks (e.g. ? mL) in these columns. ^†^The follow-up for herbal mouthwash group was up to three weeks, while the follow-up for CHX group was only two weeks.

## Abbreviations

CHX: Chlorhexidine
RCT: Randomised controlled trial
PRISMA: Preferred Reporting Items for Systematic Reviews and Meta-Analyses
PICOS: Participants-Interventions-Comparisons-Outcomes-Study design
CDSR: Cochrane Database of Systematic Reviews
CENTRAL: Cochrane Central Register of Controlled Trials
PI: Plaque Index
QHPI: Turesky modification of Quigley-Hein Plaque Index
GI: Gingival Index
MGI: Modified Gingival Index
BI: Bleeding Index
SBI: Sulcus Bleeding Index
GBI: Gingival Bleeding Index
SD: Standard deviation
WMD: Weighted mean difference
CI: Confidence interval
NR: Not reported
CPC: Cetylpyridinium chloride
PVP-I: Povidone iodine
